# State of the Art in Prostate-specific Membrane Antigen–targeted Surgery—A Systematic Review

**DOI:** 10.1016/j.euros.2023.05.014

**Published:** 2023-06-16

**Authors:** Anne-Claire Berrens, Sophie Knipper, Giancarlo Marra, Pim J. van Leeuwen, Stevie van der Mierden, Maarten L. Donswijk, Tobias Maurer, Fijs W.B. van Leeuwen, Henk G. van der Poel

**Affiliations:** a Department of Urology, Netherlands Cancer Institute–Antoni van Leeuwenhoek Hospital, Amsterdam, The Netherlands; b Interventional Molecular Imaging Laboratory, Department of Radiology, Leiden University Medical Centre, Leiden, The Netherlands; c Martini-Klinik Prostate Cancer Center, University Hospital Hamburg-Eppendorf, Hamburg, Germany; d Department of Urology, Institut Paoli-Calmettes, Marseille, France; e Urology division, Department of Surgical Sciences, Molinette Hospital, Città della Salute e della Scienza San Giovanni Battista Hospital and University of Turin, Turin, Italy; f Department of Urology, University Hospital Hamburg-Eppendorf, Hamburg, Germany; g Department of Urology, Amsterdam UMC, VU University, Amsterdam, The Netherlands; h Scientific Information Service, Netherlands Cancer Institute- Antoni van Leeuwenhoek Hospital, Amsterdam, Netherlands; i Department of Nuclear Medicine, Netherlands Cancer Institute-Antoni van Leeuwenhoek Hospital, Amsterdam, The Netherlands

**Keywords:** Fluorescence-guided surgery, Image-guided surgery, Prostate cancer, Prostate-specific membrane antigen, Radioguided surgery

## Abstract

**Context:**

Identifying malignant tissue and leaving adjacent structures undisturbed constitute an ongoing challenge in prostate cancer (PCa) surgery. Image and radioguided surgical technologies targeting the prostate-specific membrane antigen (PSMA) receptor may facilitate identification and removal of diseased tissue.

**Objective:**

To perform a systematic review of the clinical studies on PSMA-targeted surgery.

**Evidence acquisition:**

The MEDLINE (OvidSP), Embase.com, and Cochrane Library databases were searched. Identified reports were critically appraised according to the Idea, Development, Exploration, Assessment, Long-term framework criteria. The risk of bias (RoB) was assessed as per the Risk Of Bias In Non-randomized Studies—of Interventions tool. The strengths and limitations of the techniques and corresponding oncological outcomes were extracted as areas of interest. Data were reported according to the Preferred Reporting Items for Systematic Reviews and Meta-analyses guidelines.

**Evidence synthesis:**

In total, 29 reports were selected, including eight prospective studies, 12 retrospective analyses, and nine case reports, all with a high or an unclear RoB. In 72.4% of studies, PSMA targeting was achieved via radioguided surgery (RGS), predominantly using ^99m^Tc-PSMA-I&S (66.7%). Hybrid approaches that complement RGS with optical guidance are emerging. The majority of studies retrieved were pilot studies with a short follow-up. In 13 reports, salvage lymph node surgery was discussed (44.8%). In 12 more recent reports (41.4%), PSMA targeting was studied in primary PCa surgery (50.0% lymph nodes and 50.0% surgical margins), and four studied both primary and salvage surgery (13.8%). Overall, specificity was higher than sensitivity (median 98.9% and 84.8%, respectively). Oncological outcomes were discussed only in reports on the use of ^99m^Tc-PSMA-I&S in salvage surgery (median follow-up of 17.2 mo). A decline in prostate-specific antigen level of >90% ranged from 22.0% to 100.0%, and biochemical recurrence ranged from 50.0% to 61.8% of patients.

**Conclusions:**

In PSMA-targeted surgery, most studies address salvage PSMA-RGS using ^99m^Tc-PSMA-I&S. Available evidence suggests that the specificity of intraoperative PSMA targeting is higher than the sensitivity. The studies that included follow-up did not yet objectify a clear oncological benefit. Lacking solid outcome data, PSMA-targeted surgery remains investigational.

**Patient summary:**

In this paper, we review recent advances in prostate–specific membrane antigen (PSMA)-targeted surgery, which is used to help identify and remove prostate cancer. We found good evidence to suggest that PSMA targeting helps identify prostate cancer during surgery. The oncological benefits have yet to be investigated further.

## Take Home Message

We found good evidence to suggest that prostate-specific membrane antigen targeting helps identify prostate cancer during surgery. The oncological benefits have yet to be investigated further.

## 1 Introduction

During both primary and salvage prostate cancer (PCa) surgery, identifying the target PCa tissue among the surrounding healthy tissue provides a key challenge [1]. Patients are significantly more likely to have biochemical recurrence (BCR) and undergo adjuvant or early salvage cancer treatment when tumor-containing tissue remains in situ following PCa surgery. In general, surgeons rely on experience, anatomical knowledge, and the ability to correctly interpret preoperative imaging to resect PCa tissue [2]. The use of intraoperative imaging and radioguidance helps better distinguish between cancerous and healthy tissue during surgery [3], [4].

Prostate-specific membrane antigen (PSMA) is a transmembrane glycoprotein that is highly overexpressed in PCa cells and is used as the target for positron emission tomography (PET) imaging [5]. Owing to its high specificity, it is more accurate for nodal staging than magnetic resonance imaging (MRI), abdominal contrast-enhanced computed tomography (CT), or choline PET/CT, making its use increasingly common in staging of primary and recurrent PCa [5], [6]. However, the technique is less reliable for identifying small lymph node metastases (micro metastases <3 mm), and the PSMA-PET tracers are typically excreted by the kidneys, making it difficult to locate the primary cancer site [5], [7].

**Fig. 1:**
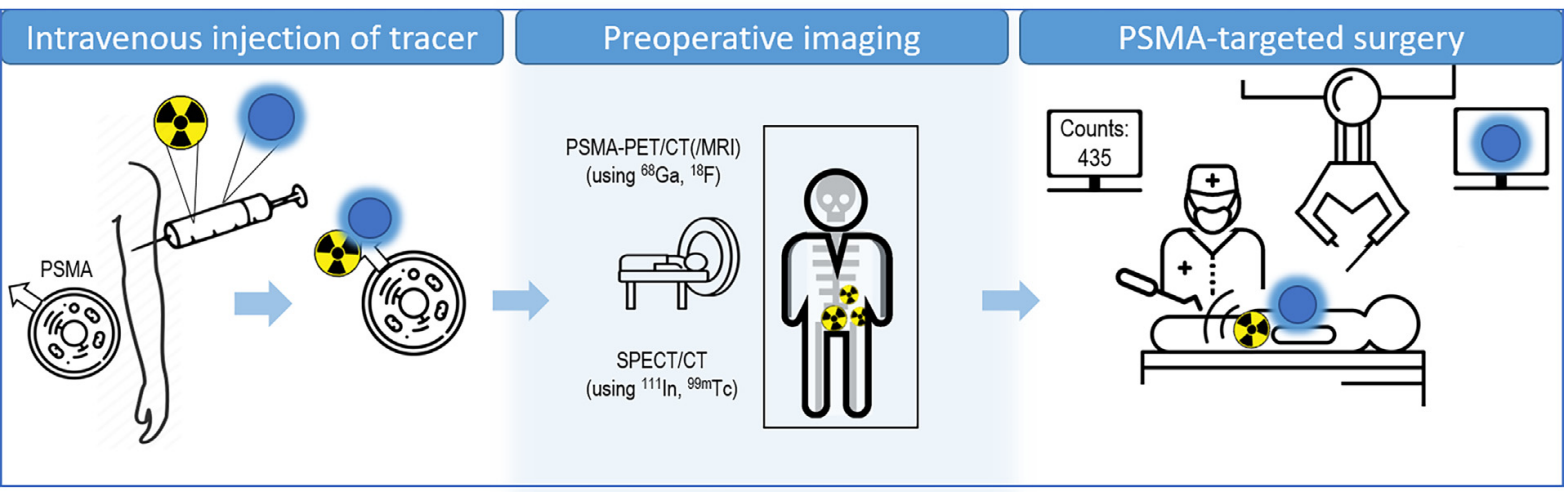
Schematic overview of clinical implementation of prostate-specific membrane antigen (PSMA)-guided surgery. Preoperative imaging can be either PSMA positron emission tomography (PET)/CT or single photon emission computed tomography (SPECT)/CT. CT = computed tomography; MRI = magnetic resonance imaging.

PSMA targeting has been proposed to extend beyond cancer diagnosis and into surgical guidance [7]. Multiple groups have explored a variety of tracer designs to realize this application, leading to the development of, for example, ^99m^Tc-PSMA–targeted radiotracers [8], [9]—tracers that support noninvasive single photon emission computed tomography (SPECT)/CT, providing a surgical roadmap, as well as allow for intraoperative image guidance (Fig. 1) [5].

As of today, different PSMA-targeting surgical approaches have been used in PCa patients. In order to provide a comprehensive overview of the techniques and initial outcome data, a systematic review of the available clinical literature was conducted.

## 2 Evidence acquisition

### 2.1 Protocol registration and search strategy

**Fig. 2:**
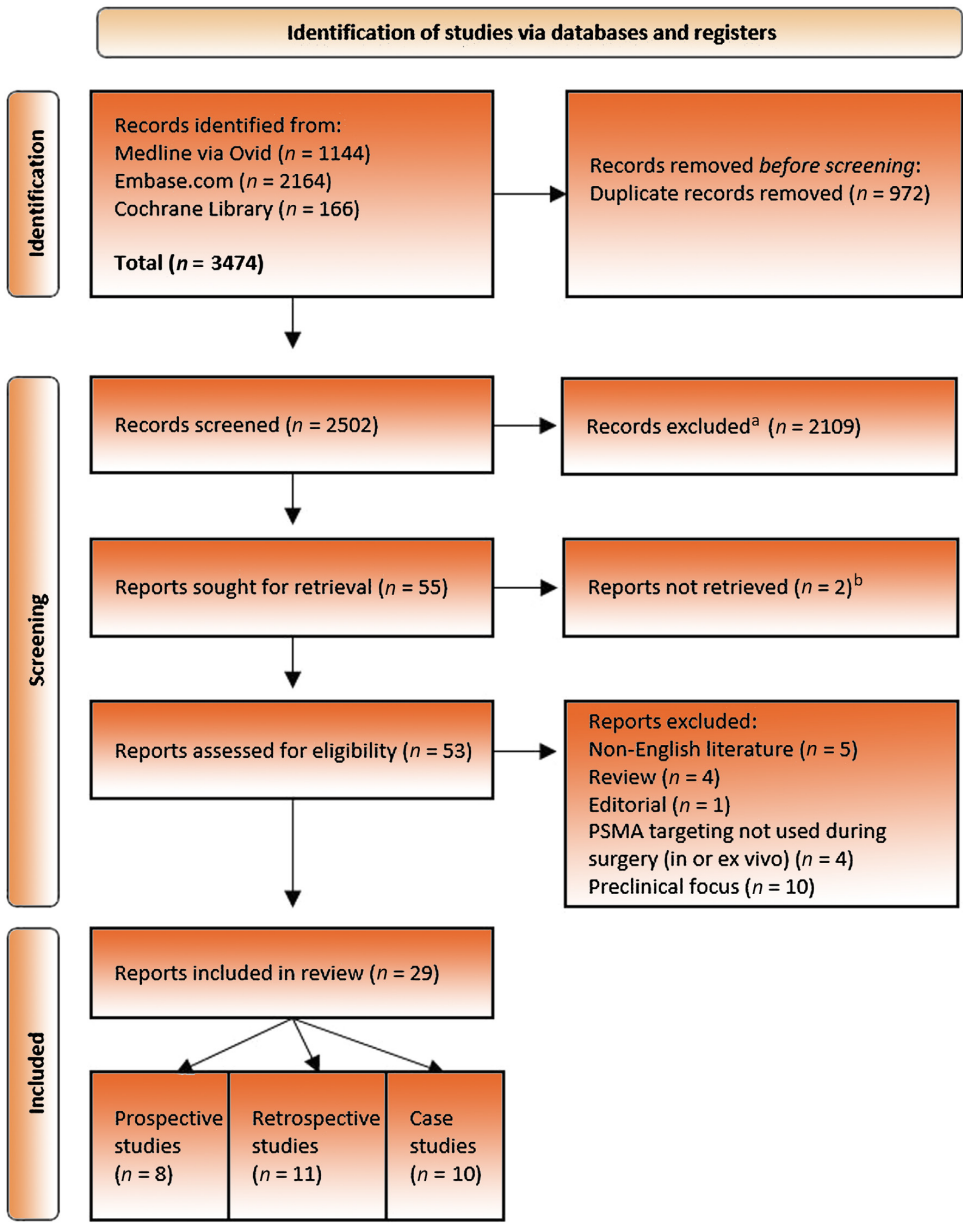
PRISMA flowchart for literature search and selection. PRISMA = Preferred Reporting Items for Systematic Reviews and Meta-analyses; PSMA = prostate-specific membrane antigen. ^a^Nonautomated. Records excluded by the author using Rayyan. ^b^Only abstract available.

The protocol was registered on PROSPERO (CRD42022304195) in January 2022. A systematic web search was conducted using MEDLINE (OvidSP), Embase.com, and Cochrane Library (Fig. 2). The search was last updated in August 2022. The search was executed with the help of an expert information specialist (S.v.d.M.) and checked by a second information specialist. Search terms can be found in the Supplementary material. Conference abstracts from Embase.com were removed based on their indexed publication type. Citation chasing was done by one person. No other methods to acquire additional reports and no other limits were used. The results were deduplicated in EndNote 20 using the method of Bramer et al. [10]. After removal of duplicates, two authors (A.C.B. and S.K.) screened all abstracts and reviewed the full-text reports for eligibility using Rayyan software [11]. Discrepancies were resolved through consensus or by consultation with a third author (G.M.). Data collection focused on demographics and surgical and oncological outcomes, and was collected by two authors in a prespecified form in Excel (Microsoft Corporation, Redmond, WA, USA). The review was reported according to the Preferred Reporting Items for Systematic Reviews and Meta-analyses (PRISMA) guidelines [12].

### 2.2 Inclusion and exclusion criteria

As the first reports on the subject of PSMA targeting in surgery became available in 2015, the studies included in this review date from 2015 to 2022. Our review incorporated research that assessed the effectiveness of intraoperative PSMA-targeted surgical guidance in directing the surgeon toward the targeted tissue in vivo or that could confirm the target through an ex vivo analysis at the back table. Surgical procedures assisted by only preoperative PSMA PET for decision-making without intraoperative or back-table guidance were excluded. Only studies that were registered with a study protocol were considered prospective. Given the dynamic growth of PSMA-targeted surgery activities, case reports were included as well. Only original English-language literature full-text reports were considered. Pure preclinical work was excluded.

### 2.3 Assessment of risks of bias

**Fig. 3:**
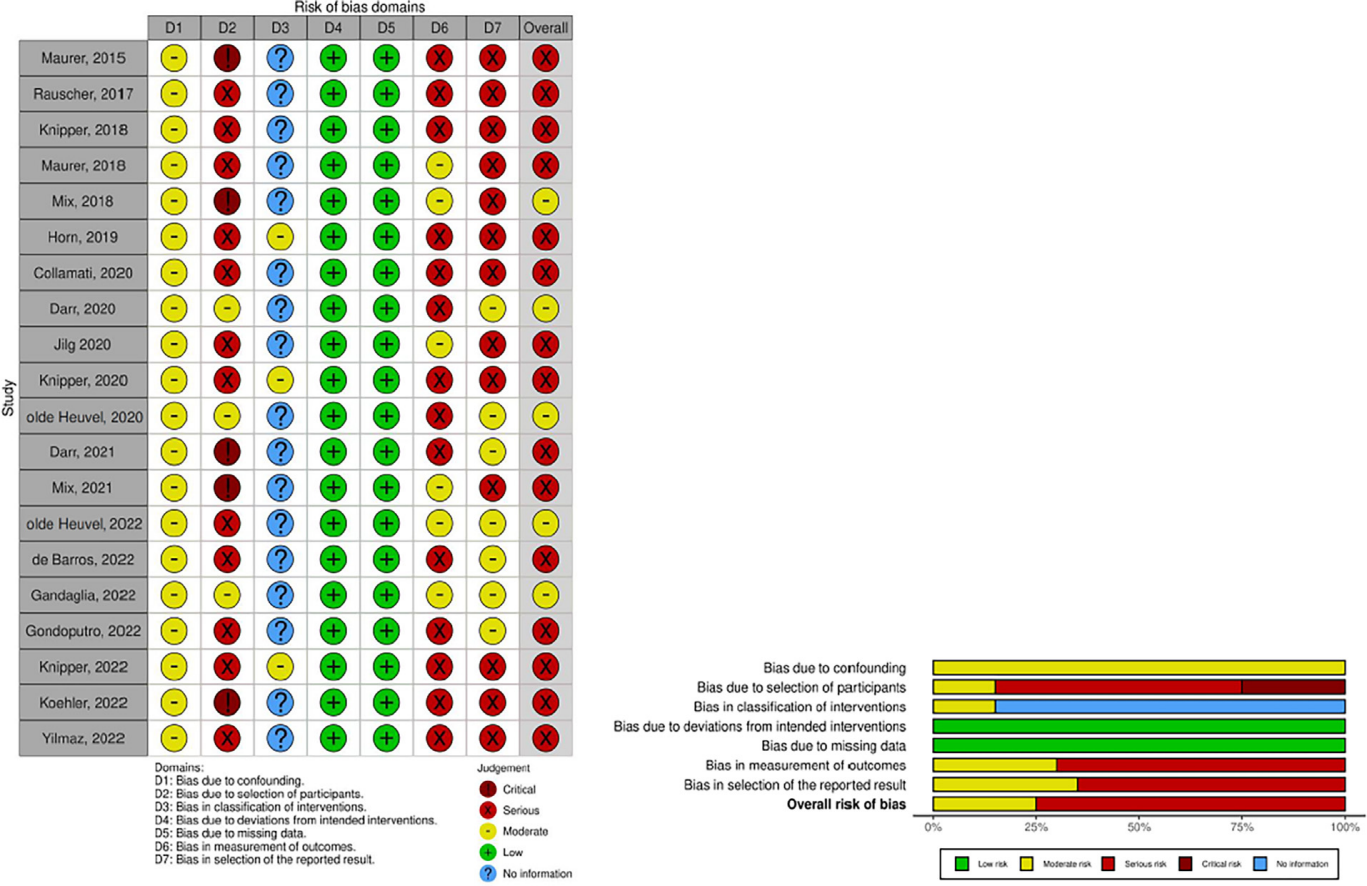
Bias table according to Risk Of Bias In Non-randomized Studies—of Interventions (ROBINS-I). (Case reports were excluded from this bias analysis.)

The studies were defined according to the Idea, Development, Exploration, Assessment, Long-term (IDEAL) framework (Table 1) [13]. The risk of bias was assessed through the Risk Of Bias In Non-randomized Studies—of Interventions(ROBINS-I) tool (Fig. 3) [14]. As the bias of case reports is not contributory, these were excluded from the assessment of the risk of bias.

**Table 1:** IDEAL framework and surgical outcomes

*Focus on lymph nodes*														
Ref	Author (year)	IDEAL framework stage	Patients (patients treated with PSMA GS)	PSMA agent, administration, type of guidance	Type of surgery	Modality used	In or ex vivo	Total targets identified				Sensitivity of surgical intervention	Specificity of surgical intervention	Metastasis size (mm) at histopathology
			*n (n)*					PSMA PET/CT (/MRI)	SPECT/CT	Intraoperatively/ex vivo	Tumor + at histopathology/total removed	% (95% CI)	% (95% CI)	Median (IQR)
[29]	Maurer (2015)	1	5	^111^In-PSMA-I&T, IV, R	Open RP + ePLND sLND	γ-probe declipse SPECT	In and ex vivo	13	–	15	15/NR	NR	NR	2.0–12.0 a
[33]	Rauscher (2017)	2a	31	^111^In-PSMA-I&T, IV, R	Open sLND	γ-probe	In and ex vivo	NR	–	54 FP = 6 FN = 4	52/145	92.3 (83.2–96.7)	93.5 (81.7–97.9)	NR
[4]	Knipper (2019)	2a	42 (13)	^99m^Tc-PSMA-I&S, IV, R	Open sLND	γ-probe	In and ex vivo	2.31^b^ (1–6)^a^	NR	NR	5^b^ (1–15)^a^/range 2–53	NR	NR	NR
[30]	Maurer (2018)	2a	31	^99m^Tc-PSMA-I&S, IV, R	Open sLND	γ-probe	In and ex vivo	44	25	46 FP = 0 FN = 12	58/132	83.6 (70.9–91.5)	100 (–)	12.0 (3.0–25.0) a
[31]	Mix (2018)	2a	6	^111^In-PSMA-617, IV, R	Open RP + ePLND sLND	γ-probe HGD	In and ex vivo	NR	NR	35 FP = 2 FN = 3	38/318 single samples	92.1 (–)	98.8 (–)	NR
[24]	Horn (2019)	2b	121	^111^In-PSMA-I&T, ^99m^Tc-PSMA-I&S, IV, R	Open sLND	γ-probe	In and ex vivo	175	NR	180	214/median 11	NR	NR	3.0 (of regions)
[15]	Collamati (2020)	1	7	^68^Ga-PSMA-11, IV, R	RA RP + ePLND	β-probe	Ex vivo	LNs: 4	–	LNs: 4 FP = 1	LNs: 3/NR	NR	NR	Smallest identified node 7 mm
[25]	Jilg (2020)	2	23 (21)	^111^In-PSMA-617, IV, R	Open RP + ePLND sLND	γ-probe HGD	In and ex vivo	87	NR	γ-Probe: 72 HGD: 79	104/864	γ-Probe: 62.1 (–) HGD: 71.2 (–)	γ-Probe: 96.3 (–) HGD: 96.9 (–)	NR
[32]	Mix (2021)	1	6	^99m^Tc-PSMA-I&S, IV, R	Open RP sLND	γ-probe	In and ex vivo	NR	NR	118	154/516	76.6 (0.69–0.83)	94.0 (0.91–0.97)	NR
[17]	de Barros (2022)	2a	20	^99m^Tc-PSMA-I&S, IV, R	RA sLND	γ-probe	In and ex vivo	21	13	19 FN = 3	21/21	86.0 (–)	100 (–)	8.4 (3.9–15.0)
[19]	Gondoputro (2022)	2a	12	^99m^Tc-PSMA-I&S, IV, R	RA RP + ePLND	γ-probe CT-guided hookwire	In and ex vivo	11	4	18 FP = 2 FN = 5	22/74	In vivo 76.0 (53.0–92.0) Ex vivo 76.0 (53.0–92.0)	In vivo 69.0 (55.0–81.0) Ex vivo 96.0 (87.0–99.0)	9.0 (6.3–11.2) Smallest <1 mm
[27]	Knipper (2022)	2b	364	^111^In-PSMA-I&T, ^99m^Tc-PSMA-I&S, IV, R	Open sLND	γ-probe	In and ex vivo	364	NR	364 FP = 21	343/NR	NR	NR	NR
[23]	Yılmaz (2022)	1	15	^99m^Tc-PSMA-I&S, IV, R	RA RP + ePLND	γ-probe	In and ex vivo	NR	NR	18	18/297	100 (–)	100 (–)	NR
[18]	Gandaglia (2022)	1	12	^99m^Tc-PSMA-I&S, IV, R	RA RP + ePLND	γ-probe	In and ex vivo	2	2	5 FP = 1 FN = 4	4/96 specimens	50.0	99.0	NR
[28]	Koehler (2023)	1	9	^99m^Tc-MIP-1404, IV, R	Open sLND	γ-probe	In and ex vivo	19	12	21	24/154	87.5	100.0	6 (2-4.5)
*Case reports*														
[41]	Schottelius (2015)	1	1	^111^In-PSMA-I&T, IV, R	Open sLND	γ-probe	In and ex vivo	NR	NR	NR	NR/NR	NR	NR	NR
[39]	Maurer (2016)	1	1	^111^In-PSMA-I&T, IV, R	Open sLND	γ-probe	In and ex vivo	1	–	1	1/NR	NR	NR	NR
[40]	Robu (2017)	1	2 (1)	^99m^Tc-PSMA-I&S, IV, R	Open RP + ePLND	γ-probe	In and ex vivo	1	–	1	1/NR	NR	NR	NR
[38]	Kratzik (2018)	1	1	^99m^Tc-PSMA-I&S, IV, R	Open sLND left sided	γ-probe	In and ex vivo	1	1	1	1/NR	NR	NR	NR
[35]	Darr (2020)	1	1	^68^Ga-PSMA-11, IV, O	Open sLND	CLI	Ex vivo	1	–	1	2/17	NR	NR	–
[42]	van Leeuwen (2019)	1	1	^99m^Tc-PSMA-I&S, IV, R	RA sLND	γ-probe	In and ex vivo	1	1	1	1/NR	NR	NR	NR
[34]	Aras (2021)	1	10 (2)	^18^F-BF3-Cy3-ACUPA, IV, O	Open RP + ePLND	Solis 525C LED illuminator with CMOS camera	Ex vivo	NR	NR	4	2/NR	NR	NR	NR
[37]	Erfani (2022)	1	1	^99m^Tc-PSMA IV, R	Open sLND	γ-probe	In and ex vivo	2	2	2	2/8	NR	NR	NR
	Author, year		Number of patients	PSMA agent, administration	Type of surgery	Modality used		Surgical margins				Sensitivity	Specificity	
								PSM intraoperatively/ex vivo		PSM at histopathology		% (95% CI)	% (95% CI)	
*Focus on prostate/local recurrence*														
[26]	Knipper (2021)	2b	40	^111^In-PSMA-I&T, ^99m^Tc-PSMA-I&S, IV, R	Open sLND	γ-probe	In and ex vivo	NR	NR	NR	NR	NR	NR	NR
[22]	Darr (2020)	1	10	^68^Ga-PSMA-11, IV, O	Open RP	CLI	Ex vivo	2		3		NR	NR	NA
[20]	olde Heuvel (2020)	1	5	^68^Ga-PSMA-11, IV, O	RA RP	CLI	Ex vivo	5 FP = 2 FN = 0		3		NR	NR	NA
[21]	olde Heuvel (2022)	1	15	^68^Ga-PSMA-11, IV, O	RA RP	CLI	Ex vivo	6 hotspots FP = NR FN = 4 hotspots		10 hotspots		NR	NR	NA
[16]	Darr (2021)	1	7	^68^Ga-PSMA-11, IV, O	Open RP	CLI + 550 nm OF	Ex vivo	3		3		NR	NR	NA
*Case reports*														
[36]	Eder (2021)	1	1	^68^Ga-PSMA-914, IV, O	RA RP	NR	In and ex vivo	1	–	1	NR	NR	NR	NA

CI =confidence interval; CLI = Cerenkov luminescence imaging; CMOS = complementary metal oxide semiconductor; CT = computed tomography; ePLND = extended pelvic lymph node dissection; FN = false negative; FP = false positive; Ga = gallium; HGD = high-purity germanium detector; IDEAL = Idea, Development, Exploration, Assessment, Long term; I&S = imaging and surgery; I&T = imaging and therapy; In = indium; IQR = interquartile range; IV = intravenous; LN = lymph node; MRI = magnetic resonance imaging; NA = not applicable; NR = not reported; O = optical guidance; OF = optical short-pass filter; PET = positron emission tomography; PSM = positive surgical margin; PSMA = prostate-specific membrane antigen; R = radioguidance; RA = robot assisted; RP = radical prostatectomy; SD = standard deviation; sLND = salvage lymph node dissection; SPECT = single photon emission computed tomography.

∼Maximum.

a Range.

b Mean (±SD).

### 2.4 Data analysis and objectives

Owing to the high heterogeneity found among the included studies in terms of different procedures, different reporting, and different definitions of outcomes, a meta-analysis was not possible. A comprehensive narrative synthesis of the included studies was performed. Descriptive statistics were used to summarize baseline characteristic data.

## 3 Evidence synthesis

### 3.1 Study quality and baseline features

**Fig. 4:**
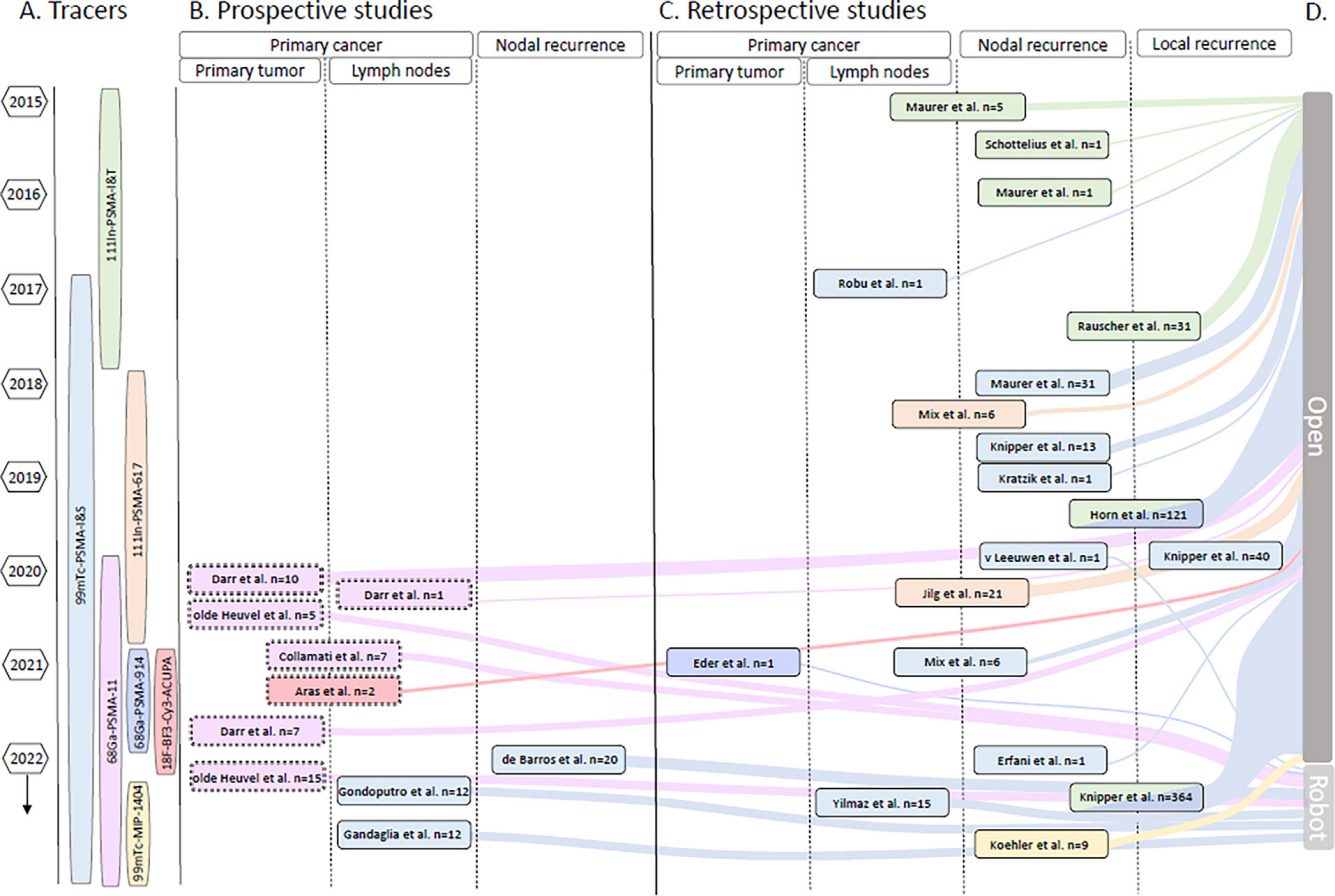
An overview of all reviewed literature. (A) All tracers chronologically aligned with year(s) of publication. (B) An overview of prospective studies. The color matches the studied tracer in A. Position on the *y* axis indicates the year of publication. Position on the *x* axis indicates the type of prostate cancer studied. (C) Overview of studies that retrospectively analyzed the data. The color matches the studied tracer in A. Position on the *y* axis indicates the year of publication. Position on the *x* axis indicates the type of prostate cancer studied. (D) Sankey diagram entwined in Figure 4B and C showing the distribution between open and robot-assisted surgical procedures by the number of patients. The color matches the tracer that was studied. PSMA = prostate-specific membrane antigen, The double outline ‘**=**’ means only ex vivo measurements were performed; the solid outline ‘**—**’ means in and ex vivo measurements were performed.

We included 29 reports on PSMA-targeted surgery (Fig. 2), of which eight were prospective studies [15], [16], [17], [18], [19], [20], [21], [22], 12 retrospective analyses [4], [23], [24], [25], [26], [27], [28], [29], [30], [31], [32], [33], and nine case reports [34], [35], [36], [37], [38], [39], [40], [41], [42] Generally, a small number of patients were included: in eight of 29 (28%) reports ≥20 patients were included, of which two (retrospective) studies included >100 patients (7%). The remaining 72% of studies can be considered small-scale pilots or first-in-man reports (Fig. 4). In the primary setting (∼41% of reports), patients included were at an intermediate or a high risk with or without nodal involvement. In the salvage setting (∼45% of reports), the included patients had recurrent disease on PSMA PET/CT (in local recurrence a maximum of one lesion and in nodal recurrence a maximum of five) and were eligible for surgery. The maximum standard uptake values of the PSMA-PET/CT scans were not taken into account. A detailed description of patients’ demographics can be found in Supplementary Table 1.

Seventeen studies (59%; including ten case reports) were considered stage 1 according to the IDEAL framework: proof of concept or first in man. These studies describe the clinical use of novel tracers or an adapted version of a previously studied tracer. Nine were considered exploring stage (stage 2a; 31%) and three were in developmental stage (stage 2b; 10%). None of the studies could be considered to be at stage ≥3 (assessment and long term; see Table 1) [13].

### 3.2 Preoperative features

Surgical resections were always guided by preoperative imaging roadmaps acquired >24 h prior to surgery (PSMA PET/CT 97% and [additional] PET/MRI 7% [28], [40]). In general, the guidance provided by PSMA PET/CT was considered leading even in cases where a SPECT/CT scan was done (see below). Fifteen (52%) of the studies reported on the outcomes of PSMA PET/CT at lesion level. Out of 473 tumor-positive lesions at histopathology, PSMA PET/CT identified 382 (81%). The smallest lesion identified on PSMA PET was 3 mm in size [4].

For the most commonly used tracers (^99m^Tc-PSMA-I&S and ^111^In-PSMA-I&T), the mean injection time to surgery was around 24 h (range 16–28 h), with a median injected activity varying between 541 and 638 MBq and between 140 and 150 MBq, respectively. Studies used ^68^Ga-PSMA-11 injected between 76 and 127 MBq [16], [20]. A detailed description of the timing and injected activity of all tracers can be found in Supplementary Table 2.

When ^111^In- or ^99m^Tc-labeled PSMA tracers were used, an additional preoperative SPECT/CT scan was performed within 24 h before surgery (in 43% of the procedures with ^111^In and in 93% with ^99m^Tc). Of the studies that included SPECT/CT, only seven reported their findings, indicating that 60 out of 133 histopathologically confirmed tumor-positive lesions (45%) could be identified by SPECT/CT. The only studies that described all lesions being identified on SPECT/CT were case reports with a maximum of two lesions on the PSMA PET/CT [37], [38], [42].

### 3.3 Radioguidance

Clinically, PSMA guidance is employed in two main approaches: radioguidance and optical guidance. The former is most frequently described (22/29 reports [76%]; ranging from one to 364 patients) and makes use of gamma/beta-emitting radioisotopes. Clinical use of gamma-emitting radioisotopes started with the use of ^111^In-PSMA-I&T, followed not shortly by studies using ^99m^Tc-PSMA-I&S and ^111^In-PSMA-617 [29], [31], [41]. In all these reports, guidance was facilitated by real-time tracing using a gamma probe. The design of the gamma probe varied depending on the surgical approach. A hand-held gamma probe for open surgery was used in 17/29 studies (59%; ranging from one to 364 patients). One study reported the combined use with a probe-based freehand SPECT scan [29]. The development of a DROP-IN gamma probe design enabled the performance of the first robotic PSMA-targeted surgery, a technique that was used in 5/29 studies (17%; ranging from one to 20 patients). In one study, a robotic DROP-IN beta probe has been employed ex vivo to confirm the presence of ^68^Ga-PSMA-11 in the prostate and nodal tissue [15].

Since 2018, all groups originally reporting the use of ^111^In-PSMA tracers (gamma emissions 171 kilo electron Volt [keV] and 245 keV; *t*_1/2_ = 2.8 d) converted to the use of ^99m^Tc-PSMA-I&S; the 141 keV gamma emission and 6 h half-life of ^99m^Tc are more compatible with the everyday clinical workflow and suffer less from background signals (details on tracer properties can be found in Supplementary Fig. 1 and Supplementary Table 3) [40]. Figure 4 shows that in 2020 ^111^In-PSMA-617 ceases to be reported on and that new ^99m^Tc-based tracers such as ^99m^Tc-MIP-1404 are still being introduced.

### 3.4 Optical guidance

A different approach of PSMA guidance relies on converting the beta-emission of tracers such as ^68^Ga-PSMA-11 into a secondary optical signal (Cerenkov Luminescence; λ_em max_ < 450 nm) that has a very limited degree of tissue penetration (<3 mm) but can be recorded in a back-table (ex vivo) dark-room environment via highly sensitive optical detectors [43]—a strategy that was used in five of 29 (17%) studies reported (37 patients).

An alternative approach to combining beta emissions and optical guidance makes the use of the so-called hybrid (radioactive and fluorescently labeled) PSMA tracers [34], [36]—a strategy that was used in 2/29 (7%) studies (three patients). Eder et al. [36] reported the use of a PSMA-11–derived hybrid molecule tracer, PSMA-914 (^68^Ga-PSMA-914), using fluorescence imaging [44], while Aras et al. [34] used ^18^F-BF3-Cy3-ACUPA in an ex vivo setting. Unfortunately, the dyes 800 CW (λ_em max_ = 789 nm [45]) and Cy3 (λ_em max_ = 565 nm) are not optimally compatible with the da Vinci Firefly endoscope (Intuitive Surgical Inc., Sunnyvale, CA, USA), which is designed for the detection of indocyanine green (λ_em max_ = 820 nm; Supplementary Table 2 and Supplementary Fig. 1). As a result, the fluorescence of ^18^F-BF3-Cy3-ACUPA was imaged only ex vivo, for neither compound was investigated if radioguidance and optical guidance could complement each other.

### 3.5 Tumor-to-background values

A comparison between tracers or modalities was difficult as the in vivo as well as ex vivo reporting of tumor-to-background ratio (TBR) was not consistent, and different cutoffs to consider a lesion positive were used [18], [28]. Mostly this was defined as at least twice the background, with different definitions of the background (fatty tissue, lymph nodes, and psoas muscle). TBR ranged from 2.1 to 8.0 for ^99m^Tc-PSMA-I&S (in vivo analysis, DROP-IN gamma probe, background: different types of tissue near the lesion) and from 10.0 to 30.0 for ^99m^Tc-MIP-1404 (ex vivo analysis, hand-held gamma probe, background: fatty tissue) [17], [28].

### 3.6 Surgical outcomes

As illustrated in Figure 4, 69% of the reports studied open (tracers used in vivo: ^111^In-PSMA-I&T, ^99m^Tc-PSMA-I&S, and ^99m^Tc-MIP-1404; ex vivo: ^68^Ga-PSMA-11 and ^18^F-BF3-Cy3-ACUPA) and 31% robot-assisted (tracers used in vivo: ^99m^Tc-PSMA-I&S and ^68^Ga-PSMA-914; ex vivo: ^68^Ga-PSMA-11) surgical interventions. Six studies (21%) focused only on the identification of margins in primary cancer (tracers used in vivo: ^68^Ga-PSMA-914; ex vivo: ^68^Ga-PSMA-11) or local recurrence (in vivo: ^99m^Tc-PSMA-I&S), two (7%) on both prostate and lymph nodes (tracers used ex vivo: ^68^Ga-PSMA-11 and ^18^F-BF3-Cy3-ACUPA), and 21 (72%) on identifying tumor-positive lymph nodes during primary or salvage lymph node dissection (LND; tracers used in vivo: ^111^In-PSMA-617, ^111^In-PSMA-I&T, ^99m^Tc-PSMA-I&S, and ^99m^Tc-MIP-1404).

In nodal surgery, the accuracy of identifying tumor-containing lymph nodes was reported in 11 studies (38%): salvage LND in seven and primary LND in four (tracers used in vivo and ex vivo: ^111^In-PSMA-617, ^111^In-PSMA-I&T, ^99m^Tc-PSMA-I&S, and ^99m^Tc-MIP-1404; tracers used only ex vivo: ^68^Ga-PSMA-11). Generally, specificity (median 96.3, interquartile range [IQR] 93.8–99.4) was higher than sensitivity (median 80.1, IQR 76–92.1). Gondoputro et al. [19] reported the only discrepancy with in vivo specificity of 69% (sensitivity of 76%; ^99m^Tc-PSMA-I&S; robotic procedure). A notable outlier was sensitivity of 50%, reported by Gandaglia et al. [18], in primary LND using ^99m^Tc-PSMA-I&S. The influence of the tracers and the approach (open/robotic) on the accuracy could not be assessed given the different preoperative patient characteristics, differences in timing of injection, differences in location of the lesions, and differences in reporting.

In vivo identified PCa lesions ranged between <1 and 25 mm in size for ^99m^Tc-PSMA-I&S, where the smallest lesion (<1 mm) was identified during robot-assisted surgery using a DROP-IN gamma probe [19]. For ^111^In-PSMA-I&T, the range was 2––12 mm. Four studies (14%) report identifying tumor-positive lesions via ^99m^Tc-PSMA-I&S that did not show on preoperative PET [18], [19], [30]. False negatives, when reported, were lesions <5 mm [17], [18], [19]. An overview of the surgical outcomes can be found in Table 1.

In prostate-focused surgery, the accuracy of identifying positive surgical margins in vivo by radioguided surgery (RGS) was described only in one patient by Gondoputro et al. [19]. They successfully removed residual cancerous tissue but advised caution due to potential urinary contamination. The ex vivo evaluation of ^68^Ga-PSMA-11 with the DROP-IN beta probe correctly identified positive surgical margins in 40% (seven patients) [15]. Ex vivo Cerenkov imaging of ^68^Ga-PSMA-11 yielded 60–83% (37 patients) agreement with histopathology [16], [20], [21], [22]. Eder et al. [36] and Aras et al. [34] describe the visibility of the fluorescent component of the tracer (in and ex vivo, respectively) but did not quantify their results.

### 3.7 (Oncological) outcomes

Between the studies, the evidence varied as shown in Table 1; no study exceeded IDEAL framework stage 2b “development.”

Follow-up data after PSMA-targeted salvage surgery were analyzed retrospectively in ten studies (34%) reporting a median follow-up of 1–25.7 mo and were provided only for ^99m^Tc-PSMA-I&S and ^111^In-PSMA-I&T. Nothing has yet been reported on the difference in oncological outcomes between these tracers.

A postoperative prostate-specific antigen (PSA) decline was reported in six of the 22 reports addressing salvage lymph node surgery [4], [23], [27], [30]. The PSA decline ranged between 67% and 100% of the patients, and a decline of >90% was reported in 22–100% (Table 2), indicating variations in treatment effectiveness. Knipper et al. [4] were the first to look at the difference in PSA decline after conventional salvage LND versus the addition of PSMA-targeted radioguidance (^99m^Tc-PSMA-I&S, 42 patients) and found a significantly better outcome for the latter. However, long term follow-up data are missing.

**Table 2:** Follow-up

Ref	Author, year	Patients (patients treated with PSMA GS)	PSMA agent, administration, type of guidance	Duration (mo)	Additional treatment after PSMA GS needed	PSA progression	BCR/BCRFS	Survival	Complications (Clavien-Dindo)			
		*n (n)*		Median (IQR)	*n* (%)	PSA at FU: median (IQR) cBR/decline: *n* (%)	*n* (%) BCRFS (mo) (95% CI)	*n* (%)	I/II *n* (%)	III *n* (%)	IV *n* (%)	V *n* (%)
*Focus on lymph nodes*												
[29]	Maurer (2015)	5	^111^In-PSMA-I&T, IV, R	NR	NR	NR	NR	NR	NR	NR	NR	NR
[33]	Rauscher (2017)	31	^111^In-PSMA-I&T, IV, R	11.1 (2.7–19.4)^a^	10 (33.0) after median of 4.1 mo	cBR: 2 (22.0) with FT 15 (75.0) without FT Decline >50%: 23 (76.7) Decline >90%: 16 (53.3)	NR	NR	6 (20.0)	4 (13.0)	0 (0.0)	0 (0.0)
[4]	Knipper (2019)	42 (13)	^99m^Tc-PSMA-I&S, IV, R	NR	NR	0.069 (<0.01–3.3) a ng/ml Decline >50% (92.0) Decline >90% (53.0)	NR	NR	NR	NR	NR	NR
[30]	Maurer (2018)	31	^99m^Tc-PSMA-I&S, IV, R	13.8 (–)	11 (35.0) after median of 3.7 mo	Decline >50%: 24 (80.0) Decline >90%: 17 (57.0)	BCR: 17 (55.0) after median of 1.9 mo	NR	12 (38.7)	1 (3.2)	0 (0.0)	0 (0.0)
[31]	Mix (2018)	6	^111^In-PSMA-617, IV, R	24 (–)	NR	at FU: 0.51 (0.03–4.85)	NR	NR	NR	NR	NR	NR
[24]	Horn (2019)	121	^111^In-PSMA-I&T, ^99m^Tc-PSMA-I&S, IV, R	NR	39 (32.2) after median of 4.6 mo	cBR: 77 (66%) Decline >50%: 88 (77.0) Decline >90%: 55 (48.0)	41.8% BCRFS of at least 12 mo No significant difference between tracers	NR	29 (24.0)	11 (9.0)	0 (0.0)	1 (1.0)
[15]	Collamati (2020)	7	^68^Ga-PSMA-11, IV, R	NR	NR	NR	NR	NR	NR	NR	NR	NR
[25]	Jilg (2020)	23 (21)	^111^In-PSMA-617 IV, R	25.7 (–)	10 (43.5)	NR	14/23 clinical progression	NR	NR	NR	NR	NR
[32]	Mix (2021)	6	^99m^Tc-PSMA-I&S, IV, R	19.4 (17.0–22.7)	6 (100.0)	At FU: 1.45 (0.15–17.1)	NR	AWD 6 (100.0)	NR	NR	NR	NR
[17]	de Barros (2022)	20	^99m^Tc-PSMA-I&S, IV, R	15.0^b^	NR	Decline >50% (67.0) Decline >90% (22.0)	BCR: 14/18 (88.0)	Overall 18/19 (94.7)	5 (26.3)	0 (0.0)	0 (0.0)	1 (5.3)
[19]	Gondoputro (2022)	12	^99m^Tc-PSMA-I&S, IV, R	13 (4–22)	6 (50.0)	pPSA 5/12 (42.0)	BCR: 2/12 (16.7)	Overall 12/12 (100.0)	1 (8.3)	0 (0.0)	0 (0.0)	0 (0.0)
[27]	Knipper (2022)	364	^111^In-PSMA-I&T, ^99m^Tc-PSMA-I&S, IV, R	No BCR: 10.8 (1.2–25.1) No treatment: 10.3 (2.3–24.0)	121 (33.2)	cBR: 165 (45.3)	BCR: 225 (61.8) BCRFS: 7.8 (5.4–10.5)	NR	94 (25.6)	23 (6.3)	1 (0.28)	0 (0.0)
[23]	Yılmaz (2022)	15	^99m^Tc-PSMA-I&S, IV, R	23.5 c (14–30) a	5 (33.3)	Decline >90% (100.0)	At 2.5 yr FU, BCRFS rate 86.7%	Overall 15/15 (100.0)	NR	0 (0.0)	0 (0.0)	0 (0.0)
[18]	Gandaglia (2022)	12	^99m^Tc-PSMA-I&S, IV, R	1	3 (25.0)	pPSA 3/12 (25.0)	NR	Overall 12/12 (100.0)	0 (0.0)	3 (25.0)	0 (0.0)	0 (0.0)
[28]	Koehler (2023)	9	^99m^Tc-MIP-1404, IV, R	NR	NR	cBR: 5 (56.0)	NR	NR	NR	NR	NR	NR
*Case reports*												
[41]	Schottelius (2015)	1	^111^In-PSMA-I&T, IV, R	NR	NR	NR	NR	NR	NR	NR	NR	NR
[39]	Maurer (2016)	1	^111^In-PSMA-I&T, IV, R	NR	0 (0.0)	<0.07 1 (100.0)	NR	Overall 1 (100.0)	NR	NR	NR	NR
[40]	Robu (2017)	2 (1)	^99m^Tc-PSMA-I&S, IV, R	NR	NR	NR	NR	NR	NR	NR	NR	NR
[38]	Kratzik (2018)	1	^99m^Tc-PSMA-I&S, IV, R	1	NR	<0.01 1 (100.0)	NR	NR	0 (0.0)	0 (0.0)	0 (0.0)	0 (0.0)
[35]	Darr (2020)	1	^68^Ga-PSMA-11, IV, O	NR	NR	NR	NR	NR	NR	NR	NR	NR
[42]	van Leeuwen (2019)	1	^99m^Tc-PSMA-I&S, IV, R	NR	NR	<0.03	NR	NR	NR	NR	NR	NR
[34]	Aras (2021)	10 (2)	^18^F-BF3-Cy3-ACUPA IV, O	NR	NR	NR	NR	NR	0 (0.0)	0 (0.0)	0 (0.0)	0 (0.0)
[37]	Erfani (2022)	1	^99m^Tc-PSMA, IV, R	NR	NR	NR	NR	NR	NR	NR	NR	NR

*Focus on prostate/local recurrence*												
[26]	Knipper (2021)	40	^111^In-PSMA-I&T, ^99m^Tc-PSMA-I&S, IV, R	24.4 (11.8–41.9)	12 (30.0)	cBR: 31 (77.5)	BCR: 22 (55.0) 23.7 (9.8–not reached)		4 (10.0)	3 (7.5)	0 (0.0)	0 (0.0)
[22]	Darr (2020)	10	^68^Ga-PSMA-11, IV, O	NR	NR	NR	NR	NR	NR	NR	NR	NR
[20]	olde Heuvel (2020)	5	^68^Ga-PSMA-11, IV, O	NR	NR	NR	NR	NR	NR	NR	NR	NR
[21]	olde Heuvel (2022)	15	^68^Ga-PSMA-11, IV, O	NR	NR	NR	NR	NR	NR	NR	NR	NR
[16]	Darr (2021)	7	^68^Ga-PSMA-11, IV, O	NR	NR	NR	NR	NR	NR	NR	NR	NR
*Case reports*												
[36]	Eder (2021)	1	^68^Ga-PSMA-914, IV, O	NR	NR	NR	NR	NR	NR	NR	NR	NR

AWD = alive with disease; BCR = biochemical recurrence, defined as PSA >0.2 ng/ml; BCRFS = BCR-free survival; cBR = complete biochemical response (PSA <0.2 ng/ml); CI = confidence interval; FT = further treatment; FU = follow-up; Ga = gallium; GS = Gleason score; I&S = imaging and surgery; I&T = imaging and therapy; In = indium; IQR = interquartile Range; IV = intravenous; NR = not reported; O = optical guidance; pPSA = precursor PSA; PSA = prostate-specific antigen; PSMA = prostate-specific membrane antigen; R = radioguidance; SD = standard deviation.

a Range.

b Maximum.

c Mean (±SD).

BCR was reported in five of 22 studies regarding salvage surgery, ranging from 50% to 61.8% of the patients with follow-ups ranging from 10.3 to 25.7 mo. One study looked at BCR in the primary setting and found a BCR of 16.7% within a median time of 13 mo (IQR 4–22) [19]. Horn et al. [24] concluded from their BCR-free survival (BCRFS) data that patients with low preoperative PSA and a single lesion on preoperative PSMA PET benefitted most from PSMA-targeted surgery. A range of 0–100% of the patient population was in need of additional treatment after PSMA-targeted surgery within a median time ranging from 1 to 25.7 mo.

Complications were classified according to Clavien-Dindo [46]. Seven of the ten studies that reported on complications report on patients with a grade I/II complication, with a percentage ranging from 8.3% to 38.7%. Five studies observed complications of grade 3 (percentage ranging from 3% to 25%), and only three patients from the total population of all studies experienced a complication of grade ≥IV [17], [24], [27]. None of the complications were ascribed to the tracer or image guidance procedure. The overall survival was again mentioned only in studies using ^99m^Tc-PSMA-I&S and ^111^In-PSMA-I&T, and was 94.7–100% after a median follow-up ranging from 1 to 23.5 mo. Details concerning follow-up and outcomes can be found in Table 2.

### 3.8 Discussion

This systematic review summarized the existing literature on PSMA-targeted surgery in PCa patients. PSMA targeting provides a promising strategy to identify PCa both pre- and intraoperatively, and it seems that we have only just begun to find out what this technique can offer. Currently, the most widely implemented approach is PSMA-RGS using ^99m^Tc-PSMA-I&S (∼50% of the studies on this topic and the study with the largest number of patients [*n* = 364]). Studies that used a conventional open surgical approach were the majority (69%) in comparison with those using robot-assisted surgery (31%).

Most studies present data on the value of PSMA-RGS in men with nodal recurrence (salvage surgery). One study suggested a benefit of PSMA-RGS versus conventional salvage LND [4]. Sensitivity for detecting nodal metastases during salvage surgery was dependent on the size and location of the lesion and ranged widely from 50% to 100% (eight trials). One of the most critical factors is selection of patients. Men with lower preoperative PSA and one lesion on imaging were most likely to benefit from RGS with a complete biochemical response rate of 45–66% in the largest series.

PSMA-PET imaging is known to be less dependable in identifying lesions under 3 mm [6], [7] This corresponds to our findings for intraoperative detection, where the median size of metastases found in this review ranged from 2 to 9 mm. Smaller nodes (<3 mm) were most frequently missed [17], [19]. The level of reporting of the correlation between RGS findings and histology in studies varied, with some studies reporting at the nodal level and others at the patient level. Moreover, whether an ex vivo analysis corresponded to intraoperative findings was not reported in all studies. This makes it difficult to compare detection rates among studies.

Tracer choice may also impact detection accuracy, a choice usually based on pharmacokinetics and pharmacodynamics as well as the sensitivity and specificity of the tracer. The studies with the largest patient groups (ranging from one to 364) included in these trials received RGS facilitated by ^99m^Tc-PSMA-I&S. The properties of ^99m^Tc-PSMA-I&S are well known, but this cannot be said for many of the other tracers, and comparative studies are lacking [47]. As ^99m^Tc-PSMA-I&S has successfully been used in both the primary and the salvage setting, it is currently the favored tracer.

The oncological benefit of PSMA-targeted surgery was studied mainly using a biochemical response as an endpoint. Looking at all the data combined, there was a large variation in PSA decline. Knipper et al. [26] showed that addition of PSMA guidance to conventional salvage LND improved PSA decline, suggesting that PSMA-RGS may improve outcome in men with recurrent nodal disease when compared with conventional surgery. Around 50% of salvage RGS patients were BCR free at a median follow-up of 13.2 mo. Hereby, a low preoperative PSA level and a single lesion on preoperative PSMA PET yielded better BCRFS after salvage RGS [24].

In six studies, PSMA-RGS was used in primary LND. Sensitivity and specificity for the detection of nodal metastases was comparable with the salvage setting, but considering the short reported follow-up, no other oncological outcome data are available. Optical cancer detection by Cerenkov or fluorescence imaging has been studied only in the primary setting. No study has proved the oncological value of PSMA-targeted surgical margin imaging. PSMA tracers that solely rely on fluorescence are in development [48], [49]. However, preclinical or first-in-man data may not immediately translate to patient care. In fact, our search worryingly suggests that thus far only two optical (hybrid) tracers mentioned in preclinical reviews (^68^Ga-PSMA-914 and ^18^F-BF3-Cy3-ACUPA) were tested clinically in two case series [8], [9], [50].

This review is limited by the retrospective nature of the majority of the included studies with an unclear overlap in numbers of patients. The included studies had a high or an unclear risk of bias, were noncomparative, lacked a standardized way of reporting outcomes, and had short follow-ups. Furthermore, the sample sizes were generally small. A direct comparison of studies is also hampered by overlapping patient populations in several studies. Only one prospective first-in-man study that presented the proof-of-concept data contained ≥20 patients so far. Based on the results from this review, it is essential that further clinical trials are conducted based on standardized methodology and proper study endpoints. In addition, consensus on what PSMA-targeted surgery should provide for wider clinical implementation is desirable.

## 4 Conclusions

Consolidation of the existing literature on PSMA-targeted guidance during surgery in PCa indicates that the most common technique used is radioactive gamma-tracing in the open salvage setting. Techniques for use in robotic surgery and the addition of optical detection possibilities are in the pipeline. Intraoperative PSMA targeting has been proved to be technically sound, but no clear oncological benefit has yet been objectified. Lacking solid outcome data, currently PSMA-targeted surgical guidance should be considered an experimental treatment. Randomized controlled studies may be considered after consensus on the optimal surgical approaches and most valid clinical endpoints.

  ***Author contributions:*** Henk G. van der Poel had full access to all the data in the study and takes responsibility for the integrity of the data and the accuracy of the data analysis.

  *Study concept and design*: Berrens, Knipper, Marra, F.W.B. van Leeuwen, van der Poel.

*Acquisition of data*: Berrens, Knipper, van der Mierden.

*Analysis and interpretation of data*: Berrens, Knipper, Marra.

*Drafting of the manuscript*: Berrens, Knipper, F.W.B. van Leeuwen, van der Poel.

*Critical revision of the manuscript for important intellectual content*: Berrens, Knipper, Marra, P.J. van Leeuwen, van der Mierden, Donswijk, Maurer, F.W.B. van Leeuwen, van der Poel.

*Statistical analysis*: None.

*Obtaining funding*: F.W.B. van Leeuwen.

*Administrative, technical, or material support*: None.

*Supervision*: F.W.B. van Leeuwen, van der Poel.

*Other*: None.

  ***Financial disclosures:*** Henk G. van der Poel certifies that all conflicts of interest, including specific financial interests and relationships and affiliations relevant to the subject matter or materials discussed in the manuscript (eg, employment/affiliation, grants or funding, consultancies, honoraria, stock ownership or options, expert testimony, royalties, or patents filed, received, or pending), are the following: None.

  ***Funding/Support and role of the sponsor:*** Fijs W.B van Leeuwen was financially supported by an Nederlandse Organisatie voor Wetenschappelijk Onderzoek (NWO) -Toegepaste en Technische Wetenschappen (TTW)-Vici (TTW BGT16141) grant and Koningin Wilhelmina Fonds voor de Nederlandse Kankerbestrijding (KWF)- Publiek Private Samenwerkingen (PPS) grant (no. 2022-PPS-14852).

  ***Acknowledgement:*** Special thanks to Maarten van Meerbeek for the input on the figures and tables regarding tracer properties.
