# Supplementary material for: State of the Art in Prostate-specific Membrane Antigen–targeted Surgery—A Systematic Review
Source: Eur Urol Open Sci. 2023 Jun 16;54:43–55. doi: 10.1016/j.euros.2023.05.014 (PMC10285550; doi:10.1016/j.euros.2023.05.014)
Supplement: Supplementary data 1 [file mmc1.docx]

**Supplementary material**

**Medline (OvidSP):**

((exp "prostatic neoplasms"/) OR ((prostat*) ADJ3 (cancer* OR neoplas* OR carcinom* OR adenocarcinoma* OR malignan* OR tumor* OR tumour* OR sarcoma* OR lymphom* OR oncolog* OR metastas*) OR PCa).ti,ab,kf.) AND (("gallium 68 PSMA-11" OR "177Lu-EB-PSMA-617" OR "(18)F-PSMA-11" OR "PSMA-617" OR "18F-JK-PSMA-7" OR "89Zr-Df-IAB2M" OR "68Ga-DKFZ-PSMA-11" OR "177Lu-PSMA-617" OR "PSMA-1007" OR "PSMA-BCH").nm. OR (PSMA OR "prostate specific membrane antigen*" OR "PSM antigen*" OR "gallium gozetotide GA-68" OR "89Zr-Df-IAB2M" OR "vipivotide tetraxetan").ti,ab,kf.) AND (exp "Surgical Procedures, Operative"/ OR exp "Prostatic Neoplasms"/su OR (surger* OR surgical* OR prostatectom* OR salvage OR ("lymph node*" ADJ3 (dissection* OR biops* OR excision*)) OR LND OR PLND OR ePLND OR sLND).ti,ab,kf.)

**Embase.com:**
(('prostate tumor'/exp) OR (((prostat*) NEAR/3 (cancer* OR neoplas* OR carcinom* OR adenocarcinoma* OR malignan* OR tumor* OR tumour* OR sarcoma* OR lymphom* OR oncolog* OR metastas*)) OR PCa):ti,ab,kw) AND (('prostate specific membrane antigen'/exp OR 'gallium gozetotide ga 68'/de OR 'vipivotide tetraxetan lutetium lu 177'/de OR 'vipivotide tetraxetan'/de) OR (PSMA OR "prostate specific membrane antigen*" OR "PSM antigen*" OR "gallium gozetotide GA-68" OR "89Zr-Df-IAB2M" OR "vipivotide tetraxetan"):ti,ab,kw) AND (surgery/exp OR (surger* OR surgical* OR prostatectom* OR salvage OR (("lymph node*" NEAR/3 (dissection* OR biops* OR excision*)) OR LND OR PLND OR ePLND OR sLND)):ti,ab,kw)

**Cochrane library:**

#1 MeSH descriptor: [Prostatic Neoplasms] explode all trees

#2 ((prostat*) NEAR/3 (cancer* OR neoplas* OR carcinom* OR adenocarcinoma* OR malignan* OR tumor* OR tumour* OR sarcoma* OR lymphom* OR oncolog* OR metastas*)):ti,ab,kw OR (PCa):ti,ab,kw

#3 #1 OR #2

#4 (PSMA OR "prostate specific membrane antigen*" OR "PSM antigen*" OR "gallium gozetotide GA-68" OR "89Zr-Df-IAB2M" OR "vipivotide tetraxetan"):ti,ab,kw

#5 MeSH descriptor: [Surgical Procedures, Operative] explode all trees

#6 MeSH descriptor: [Prostatic Neoplasms] explode all trees and with qualifier(s): [surgery - SU]

#7 (surger* OR surgical* OR prostatectom* OR salvage):ti,ab,kw OR ("lymph node*" NEAR/3 (dissection* OR biops* OR excision*)):ti,ab,kw OR (LND OR PLND OR ePLND OR sLND):ti,ab,kw

#8 #5 OR #6 OR #7

#9 #3 AND #4 AND #8

Supplementary data 1.

Full search on Medline, Embase.com and Cochrane library

| ref | **Author, year** | **Number of patients** | **Age** | **Type Pca** | **Preoperative PSA*** | **ISUP** | | | | | **Pathological T-stage** | | | |
| --- | --- | --- | --- | --- | --- | --- | --- | --- | --- | --- | --- | --- | --- | --- |
|  |  | **n (n PSMA guidance)** | **Years Median (IQR)** |  | **ng/ml Median(IQR)** | **1 n (%)** | **2 n (%)** | **3 n (%)** | **4 n (%)** | **5 n (%)** | **2 n (%)** | **3a n (%)** | **3b n (%)** | **4 n (%)** |
| 31 | Maurer,  2015 | 5 | 75 (64-75)^^ | Recurrent Disease or primary N+ | 2.45 (0.42-244)^^ | 1 (20.0) | 1 (20.0) | 1 (20.0) | 1(20.0) | 1 (20.0) | 2 (40.0) | 1(20.0) | 1(20.0) | 1(20.0) |
| 25 | Rauscher,  2017 | 31 | 68.2 (60.5-73.5)^^ | Recurrent disease | 1.3 (0.57-2.53) | 5 (16.1) | 15 (48.5) | | 10 (32.3) | | 12 (38.7) | 16 (51.6) | | 1 (3.2) |
| 4 | Knipper,  2018 | 42 (13) | 61.8' (52–76)^^ | Recurrent disease | 3.5' (0.15-16.3)^^ | 0 (0.0) | 11 (84.6 | | 2 (15.4) | | 8 (61.5) | 5 (38.5) | | |
| 32 | Maurer,  2018 | 31 | 66.7 (60.5-73.5) | Recurrent disease | 1.13 (0.71-2.35) | 4 (12.9) | 8 (25.8) | 7 (22.6) | 5 (16.1) | 7 (22.6) | 14 (45.2) | 16 (51.6) | | |
| 33 | Mix,  2018 | 6 | 71 (65-77) | Recurrent disease or primary N+ | 7.03 (0.93 -12.16) | 0 (0.0) | 0 (0.0) | 3 (50.0) | 2 (33.3) | 1 (16.7) | NR | NR | NR | NR |
| 26 | Horn,  2019 | 121 | 70 (63-74) | Recurrent disease | 1.13 (0.53-2.16) | 13 (11.0) | 48 (48.0) | | 44 (36.0) | | 12 (38.7) | 16 (51.6) | | 1 (3.2) |
| 15 | Collamati,  2020 | 7 | 63 (55-71) | Primary high-risk | 5.3 (4.4-8.3) | 0 (0.0) | 0 (0.0) | 0 (0.0) | 4 (0.57) | 3(0.43) | NR | NR | NR | NR |
| 22 | Darr,  2020 | 10 | 72 (63-81)^^ | Primary high-risk | 9.04 (3.3-77.7)^^ | 0 (0.0) | 2 (20.0) | 3 (30.0) | 1 (10.0) | 4(40.0) | 2 (20.0) | 5 (50.0) | 3 (30.0) | 0 (0.0) |
| 27 | Jilg,  2020 | 23 (21) | 67(52–78)^^ | Recurrent disease or primary N+ | 1.8 (0.03–56.2)^^ | 0 (0.0) | 0 (0.0) | 4 (17.0) | 7 (31.0) | 12 (52.0) | NR | NR | NR | NR |
| 28 | Knipper,  2020 | 40 | 67 (63-67) | Recurrent disease or Primary cN+ | 0.9 (0.5-1.7) | 7 (17.5) | 12 (30.0) | 6 (15.0) | 7 (17.5) | 7 (17.5) | 18 (45.0) | 15 (37.5) | 7 (17.5) | 0 (0.0) |
| 20 | olde Heuvel,  2020 | 5 | 67 (60.5-72) | Primary high-risk | 6.4 (4.85-19.1) | 0 (0.0) | 0 (0.0) | 0 (0.0) | 3 (60.0) | 2 (40.0) | 2 (40.0) | 2 (40.0) | 1 (20.0) | 0 (0.0) |
| 24 | Mix,  2021 | 6 | 64 (54.8-67.3) | Recurrent Disease  or primary cN+ | 33.3 (4.6-64.4) | 0 (0.0) | 0 (0.0) | 2 (33.3) | 3 (50.0) | 1 (16.7) | NR | NR | NR | NR |
| 21 | olde Heuvel,  2022 | 15 | 67 (64-72.5) | Primary high-risk | 7.9 (5.2-9.25) | 0 (0.0) | 1 (6.7) | 2 (13.3) | 7 (46.7) | 5(33.3) | 4 (26.7) | 6 (40.0) | 5 (33.4) | 0 (0.0) |
| 16 | Darr,  2021 | 7 | 66 (59- 69) | Primary high-risk | 12.0 (5.6-16.0) | 0 (0.0) | 1 (14.3) | 2 (28.6) | 2 (28.6) | 2 (28.6) | 5 71.0) | 2 (29.0) | 0 (0.0) | 0 (0.0) |
| 17 | de Barros,  2022 | 20 | 68 (66-72) | Recurrent disease | 1.02 (0.46-2.43) | NR | NR | NR | NR | NR | NR | NR | NR | NR |
| 19 | Gondoputro,  2022 | 12 | 68 (57-69) | Primary high-risk | 9.15 (6.0-21.2) | 0 (0.0) | 0 (0.0) | 0 (0.0) | 3 (25.0) | 9 (75.0) | 6 (50.0) | 1 (8.3) | 3 (25.0) | 0 |
| 29 | Knipper,  2022 | 364 | 67 (62-71) | Recurrent disease | 1.0 (0.5-1.9) | 27 (7.2) | 96 (26.0) | 127 (35.0) | 40 (11.0) | 60 (16.0) | 145 (40.0) | 105 (29.0) | 107 (29.0) | 0 (0.0) |
| 23 | Yilmaz,  2022 | 15 | 63.3' (56-74)^^ | Intermediate -/ high-risk | 40.9' (4-309)^^ | 0 (0.0) | 3 (20.0) | 4 (26.7) | 1 (26.7) | 7 (46.7) | NR | NR | NR | NR |
| 18 | Gandaglia,  2022 | 12 | 70 (66-71) | Intermediate-/high-risk | 8.7 (4.8-15.5) | 0 (0.0) | 1 (8.3) | 5 (42.0) | 4 (33.0) | 2 (17.0) | 0 (0.0) | 8 (66.7) | 4 (33.3) | 0 (0.0) |
| 30 | Koehler,  2022 | 9 | 62 (61 – 67) | Recurrent disease | 0.74 (0.41 – 1.54) | 0 (0.0) | 1 (11.1) | 5 (55.5) | 2 (22.2) | 0 (0.0) | 2 (22.2) | 3 (33.3) | 4 (44.4) | 0 (0.0) |
|  | **Case reports** |  |  |  |  |  |  |  |  |  |  |  |  |  |
| 41 | Schottelius,  2015 | 1 | 51 | Recurrent disease | 63 | 0 (0.0) | 0 (0.0) | 0 (0.0) | 0 (0.0) | 1 (100.0) | NR | NR | NR | NR |
| 39 | Maurer,  2016 | 1 | 63 | Recurrent disease | 0.23 | 0 (0.0) | 0 (0.0) | 0 (0.0) | 0 (0.0) | 1 (100.0) | 0 (0.0) | 1 (100.0) | 0 (0.0) | 0 (0.0) |
| 40 | Robu,  2017 | 2 (1) | 72 | Intermediate risk | 13 | 0 (0.0) | 0 (0.0) | 1 (100.0) | 0 (0.0) | 0 (0.0) | 1 (100.0) | 0 (0.0) | 0 (0.0) | 0 (0.0) |
| 38 | Kratzik,  2018 | 1 | 78 | Recurrent disease | 13.1 | 0 (0.0) | 0 (0.0) | 0 (0.0) | 0 (0.0) | 1 (100.0) | 1 (100.0) | 0 (0.0) | 0 (0.0) | 0 (0.0) |
| 35 | Darr,  2020 | 1 | 68 | Primary cN+ | NR | 0 (0.0) | 0 (0.0) | 1 (100.0) | 0 (0.0) | 0 (0.0) | 1 (100.0) | 0 (0.0) | 0 (0.0) | 0 (0.0) |
| 42 | van Leeuwen,  2020 | 1 | NR | Recurrent disease | 0.58 | NR | NR | NR | NR | NR | NR | NR | NR | NR |
| 34 | Aras,  2021 | 10 (2) | 66' (57-74)^^ | Intermediate -/ high-risk | 31' (0.25-199)^^ | 0 (0.0) | 1 (10.0) | 4 (40.0) | 3 (30.0) | 0 (0.0) | 3 (30.0) | 6(60.0) | | 0 (0.0) |
| 36 | Eder,  2021 | 1 | 71 | Primary high-risk | 7 | 0 (0.0) | 0 (0.0) | 0 (0.0) | 0 (0.0) | 1 (100.0) | NR | NR | NR | NR |
| 37 | Erfani,  2022 | 1 | 75 | Recurrent disease | 0.3 | NR | NR | NR | NR | NR | NR | NR | NR | NR |

Supplementary Table 1. Table on study features and baseline patient characteristics. *Preoperative PSA is the value before Prostate Specific Membrane Antigen(PSMA)-targeted surgery, not primary surgery. '=Mean (± SD), ^^ =Range, ISUP= International Society of Urological Pathology, IQR=Interquartile Range, NR = not reported, Pca=Prostate cancer, PSA=Prostate specific antigen.

| Ref | **Author, year** | **Number of patients** | **PSMA agent,  administration,  type of guidance** | **Injection time to surgery** | **Injected activity MBq** | **Preoperative  imaging** |
| --- | --- | --- | --- | --- | --- | --- |
|  | **Focus on lymph nodes** |  |  | **Median (IQR)** | **Median (IQR)** |  |
| 31 | Maurer,  2015 | 5 | 111In-PSMA-I&T,  IV, R | 24h | 146' (110-169)^^ | 68Ga‑PSMA‑PET/CT |
| 25 | Rauscher,  2017 | 31 | 111In-PSMA-I&T,  IV, R | 22.9' ± 3 h (16.7–28.0)^^ | 150' (86-298)^^ | 68Ga‑PSMA‑PET/CT |
| 4 | Knipper,  2018 | 42 (13) | 99mTc-PSMA-I&S,  IV, R | NR | NR | 68Ga‑PSMA‑PET/CT  + SPECT/CT |
| 32 | Maurer,  2018 | 31 | 99mTc-PSMA-I&S,  IV, R | 19.7h' (15.8-24.9)^^ | 571' (221-857)^^ | 68Ga‑PSMA‑PET/CT SPECT/CT |
| 33 | Mix,  2018 | 6 | 111In-PSMA-617, IV, R | 48h | 108.5 (104-113) | 68Ga‑PSMA‑PET/CT SPECT/CT |
| 26 | Horn,  2019 | 121 | 111In-PSMA-I&T  99mTc-PSMA-I&S,  IV, R | 24h | NR | 68Ga‑PSMA‑PET/CT SPECT/CT |
| 15 | Collamati,  2020 | 7 | 68Ga-PSMA-11,  IV, R | 150 (120-172.5) | 68 (63.5–82) | 18F-DCFPyl-PSMA-PET/CT |
| 27 | Jilg,  2020 | 23 (21) | 111In-PSMA-617 IV, R | 44' ± 10h | 110' ± 14 | 68Ga‑PSMA‑PET/CT SPECT/CT |
| 24 | Mix,  2021 | 6 | 99mTc-PSMA-I&S, IV, R | 24h | 638' (537-692)^^ | 68Ga‑PSMA‑PET/CT  18F‑PSMA‑PET/CT SPECT/CT |
| 17 | de Barros,  2022 | 20 | 99mTc-PSMA-I&S, IV, R | 19-23h^^ | 541 (526–578) | 68Ga‑PSMA‑PET/CT  18F‑PSMA‑PET/CT SPECT/CT |
| 19 | Gondoputro,  2022 | 12 | 99mTc-PSMA-I&S, IV, R | 18h | 500 | 68Ga‑PSMA‑PET/CT SPECT/CT |
| 29 | Knipper,  2022 | 364 | 111In-PSMA-I&T  99mTc-PSMA-I&S,  IV, R | NR | NR | 68Ga‑PSMA‑PET/CT SPECT/CT |
| 23 | Yilmaz,  2022 | 15 | 99mTc-PSMA-I&S, IV, R | 17' ± 2h | 630' (555-770)^^ | 68Ga‑PSMA‑PET/CT SPECT/CT |
| 18 | Gandaglia,  2022 | 12 | 99mTc-PSMA-I&S, IV, R | NR | 735 (731-738) | 68Ga-PSMA-11 PET/CT SPECT/CT |
| 30 | Koehler,  2022 | 9 | 99mTc-MIP-1404,  IV, R | 20.8h 30 – 24.0 | 747 (710 – 764) | 68Ga-PSMA I&T PET/MRI SPECT/CT |
|  | **Case reports** |  |  |  |  |  |
| 41 | Schottelius, 2015 | 1 | 111In-PSMA-I&T, IV, R | 24h | 155 | 68Ga‑PSMA‑PET/CT SPECT/CT |
| 39 | Maurer, 2016 | 1 | 111In-PSMA-I&T, IV, R | 24h | NR | 68Ga-PSMA-PET/CT |
| 40 | Robu, 2017 | 2 (1) | 99mTc-PSMA-I&S, IV, R | 16h | 497 (-) | 68Ga‑PSMA‑PET/MRI SPECT/CT |
| 38 | Kratzik, 2018 | 1 | 99mTc-PSMA-I&S, IV, R | 18h | 800 (-) | 68Ga‑PSMA‑PET/CT SPECT/CT |
| 35 | Darr, 2020 | 1 | 68Ga-PSMA-11,  IV, O | 234min | 116 (-) | 68Ga-PSMA-11 PET/CT |
| 42 | van Leeuwen, 2020 | 1 | 99mTc-PSMA-I&S, IV, R | 24h | NR | 68Ga-PSMA-11 PET/CT SPECT/CT |
| 34 | Aras, 2021 | 10 (2) | 18F-BF3-Cy3-ACUPA IV, O | 24h | 6.5 '( ± 3.2 mCi) | [18F]-BF3-Cy3-ACUPA PET/CT + PET/MRI |
| 37 | Erfani, 2022 | 1 | 99mTc-PSMA IV, R | NR | 20 mCi | 68Ga‑PSMA‑PET/CT SPECT/CT |
|  | **Author, year** | **Number of patients** | **PSMA agent,  administration** | **Injection time to surgery** | **Injected activity MBq** | **Preoperative  imaging** |
|  | **Focus on prostate/local recurrence** |  |  | **Median (IQR)** | **Median (IQR)** |  |
| 28 | Knipper, 2020 | 40 | 111In-PSMA-I&T  99mTc-PSMA-I&S,  IV, R | 24h | NR | 68Ga‑PSMA‑PET/CT  18F-PSMA PET/CT |
| 22 | Darr, 2020 | 10 | 68Ga-PSMA-11,  IV, O | 223min(153-328)^^ | 119' (95-202^^ ) | 68Ga‑PSMA‑PET/CT |
| 20 | Olde Heuvel, 2020 | 5 | 68Ga-PSMA-11,  IV, O | 73' ± 14 min | 76 (66.5-103) | 68Ga‑PSMA‑PET/CT |
| 21 | Olde Heuvel, 2022 | 15 | 68Ga-PSMA-11,  IV, O | 70'min (44-105)^^ | 69' (23-121)^^ | 68Ga‑PSMA‑PET/CT |
| 16 | Darr, 2021 | 7 | 68Ga-PSMA-11,  IV, O | 316min (283-331) | 127 (116-179) | 68Ga-PSMA-PET/CT |
|  | **Case reports** |  |  |  |  |  |
| 36 | Eder, 2021 | 1 | 68Ga-PSMA-914, IV, O | 1h | NR | 68Ga-PSMA-914-PET/CT |

Supplementary Table 2. Table on preoperative imaging features.' =Mean (± SD), ~=maximum, ^^ =Range, Ga=Gallium, I&S=Imaging and surgery, I&T=Imaging and therapy, In=Indium, IQR=Interquartile Range, IV=Intravenous, MBq= Megabecquerel, mCi=milliCurie, NR= Not reported, O= Optical guidance, R = Radioguidance, Tc= Technetium.

Supplementary Figure 1. Figure on chemical structures of PSMA-tracers for PSMA-targeted surgery.


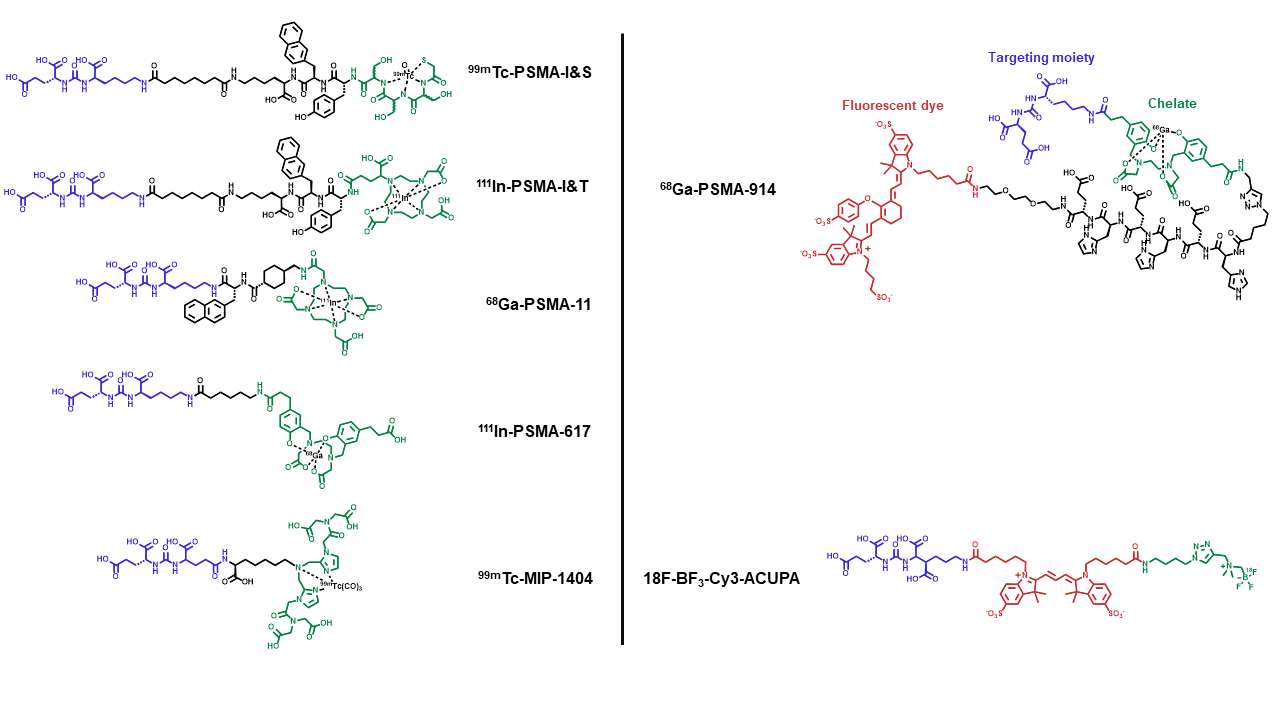


|  | **Injection** | **Preparation Time** | **Half-life** | **Radiochemical purity** | **IC50 [nM]** | **Fraction of protein-bound tracer (human plasma)** | **Stability** | **Clearance pathway** | **λ_em max_ (nm)** |
| --- | --- | --- | --- | --- | --- | --- | --- | --- | --- |
| **111In-PSMA-I&T** | IV | 10 min (labeling) | 2.8 d |  | 7.5 ± 1.5 | 83% | 5-7 days | Renal | NA |
| **111In-PSMA-617** | IV | 30 min (synthesis) | 2.81 d (111In) | 100.0±0.0% | Kd=5.4±0.8 nM | NR | NR | Renal | NA |
| **99mTc-PSMA-I&S** | IV | 90 min | 27h | 98% | 39.7 ± 1.2 | 94% | for up to 6h | Renal and hepatobilliary | NA |
| **68Ga-PSMA-11** | IV | 15 min (synthesis) | 68 min | >99% | 7.5 ± 2.2 | NR | NR | Renal, minimal lung and spleen | 450 for Cerenkov |
| **18F-BF3-Cy3-ACUPA** | IV | 25 min (labeling) | 18 min | 31-14% decay uncorrected | NR | NR | NR | Renal, minimal hepatic accumulation | 565 |
| **68Ga-PSMA-914** | IV | 10 min (labeling) | 68 min | >99% | 35.54+2.94 | NR | NR | Renal, minimal splenic clearance | 789 |
| **99mTc-MIP-1404** | IV | NR | 13.2 | 95% | Kd = 0.75 ± 0.32 nM | NR | NR | Renal and minimal hepatobilliary | NA |

Supplementary Table 3. Table on chemical properties of Prostate Specific Membrane Antigen (PSMA)-tracers for PSMA-targeted surgery. Ga=Gallium, IC50 = Half maximal inhibitory concentration, I&S=Imaging and surgery, I&T=Imaging and therapy, In=Indium, IV=Intravenous, Kd= Equilibrium dissociation constant, λem max = maximum emission wavelength**,** mCi=milliCurie, nm = nanometer, [nM] = nano molar concentration, NR= Not reported, Tc= Technetium.
